# Supplementary material for: Let’s just ask them. Perspectives on urban dwelling and air quality: A cross-sectional survey of 3,222 children, young people and parents
Source: PLOS Glob Public Health. 2023 Apr 13;3(4):e0000963. doi: 10.1371/journal.pgph.0000963 (PMC10101632; doi:10.1371/journal.pgph.0000963)
Supplement: S1 Appendix — (DOCX) [file pgph.0000963.s001.docx]

# S1 Appendix: Most successful advertisement for each target city


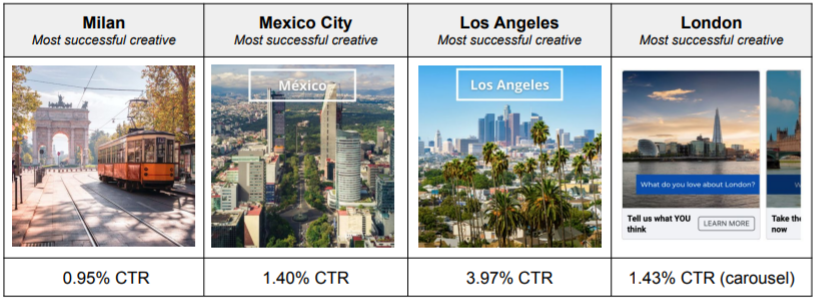

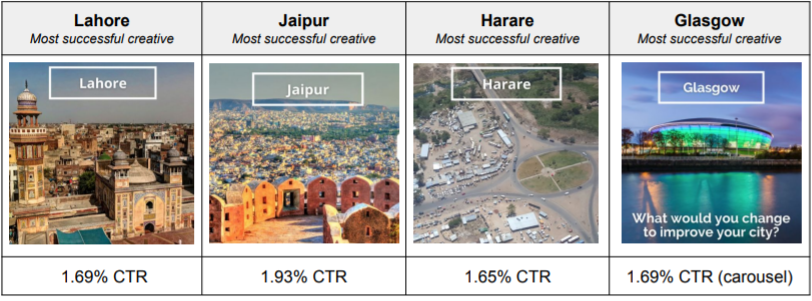

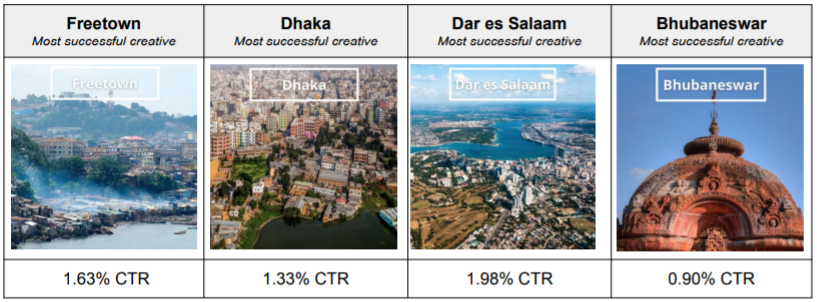

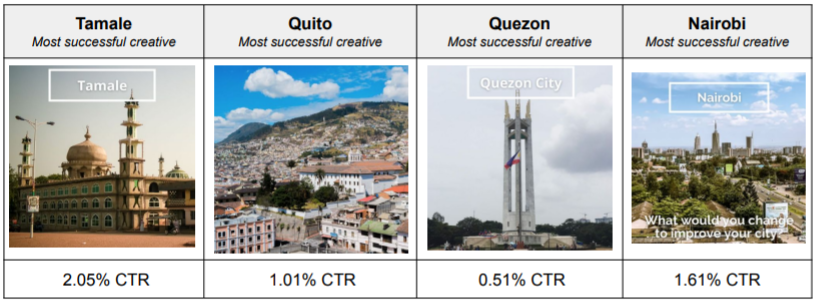


Photographs republished from Canva.com under a CC BY license, with permission from Canva.com, original copyright 2022 (https://www.canva.com/policies/content-license-agreement
