# Supplementary material for: Let’s just ask them. Perspectives on urban dwelling and air quality: A cross-sectional survey of 3,222 children, young people and parents
Source: PLOS Glob Public Health. 2023 Apr 13;3(4):e0000963. doi: 10.1371/journal.pgph.0000963 (PMC10101632; doi:10.1371/journal.pgph.0000963)
Supplement: S2 Appendix — (DOCX) [file pgph.0000963.s002.docx]

# S2 Appendix: Detailed methods for population recruitment, pretesting methods, and data handling Population Recruitment

Recruitment advertisements were targeted at people living in 16 cities in the respective languages: London (English), Glasgow (English), Milan (Italian), Quezon City (English), Los Angeles (English), Nairobi (Swahili/Sheng), Quito (Spanish), Jaipur (Hindi and Urdu), Bhubaneswar (Hindi), Dar es Salaam (Swahili/Sheng), Tamale (English), Lahore (Urdu), Dhaka (Bangla), Free Town (English), Mexico City (Spanish), Harare (English). These cities were not randomly selected, rather chosen due to existing connection to the CCC project (London, Nairobi, and Harare) or as the hosts of the 2021 COP26 Summit (Glasgow and Milan). The additional cities were chosen to reflect a variety of global settings, population sizes, and levels of development in both the Global North and South. All 16 cities were used in the co-benefits analysis completed by the CCC team, to quantify the effects of reducing urban AP on child health.

Respondents were recruited to the survey through promoted social media advertisements that targeted young people or parents in the 16 target locations, based on location and demographic data held by the social media platforms (22). Recruitment advertisements were co-developed with a specialist social media marketing agency (23), and were designed to appeal to a wide variety of young people (not just those already concerned about urban/environmental health) using a variety of short messages and images (S1 Appendix). The advertisements were pre-tested to optimise engagement for each target group and location based on clicks (rather than survey completes), to reduce the risk of introducing additional responder bias beyond that already inherent due to variable completion rates. Several adjustments were made throughout the campaign to attempt to limit bias in the sample. To identify more potential respondents from less represented areas, location radius was increased for cities with a higher cost per click and adverts which produced the lowest click-through rates were refreshed with new imagery. To reduce over representation in Tamale, Harare, and Dhaka advertisements were paused in these locations on October 5^th^, 2021 as they had produced the largest volume of eligible and consenting responses at that time. Informed consent, developed with reference to guidelines in *The Global Kids Online Research Toolkit* (24), was sought from all respondents in a relevant language before they were included into the final sample.

**Pretesting Methods**

The English-language survey instrument was pre-tested in two phases to evaluate its reliability and validity and ensure data quality. Within each phase of pre-testing, feedback on the survey instrument was collected using a Google Form (25), in parallel time with the completion of the survey instrument. Feedback was integrated into the draft Typeform survey instrument after each phase of pre-testing.

The reliability of the English-language survey instrument was further tested using two different scoring systems for readability and interpretation. The survey scored 82.3 out of 100 for the Flesch Reading Ease score[^[1]^](https://euc-word-edit.officeapps.live.com/we/wordeditorframe.aspx?new=1&ui=en%2DUS&rs=en%2DUS&wopisrc=https%3A%2F%2Flshtm.sharepoint.com%2Fsites%2FCCC_Group%2F_vti_bin%2Fwopi.ashx%2Ffiles%2Fabe8096bc52942738c40e63e20c428c5&wdenableroaming=1&mscc=1&hid=E08E9CA0-2057-6000-48B7-7F9B4CAE206B&wdorigin=Other&jsapi=1&jsapiver=v1&newsession=1&corrid=585438e0-6afc-4938-8831-11bc73244cd8&usid=585438e0-6afc-4938-8831-11bc73244cd8&sftc=1&cac=1&mtf=1&sfp=1&wdredirectionreason=Unified_SingleFlush&rct=Normal&ctp=LeastProtected#_ftn1) which indicates good overall readability. To assess the ease of interpretation for young people who have varying language comprehension levels, the CEFR Level[^[2]^](https://euc-word-edit.officeapps.live.com/we/wordeditorframe.aspx?new=1&ui=en%2DUS&rs=en%2DUS&wopisrc=https%3A%2F%2Flshtm.sharepoint.com%2Fsites%2FCCC_Group%2F_vti_bin%2Fwopi.ashx%2Ffiles%2Fabe8096bc52942738c40e63e20c428c5&wdenableroaming=1&mscc=1&hid=E08E9CA0-2057-6000-48B7-7F9B4CAE206B&wdorigin=Other&jsapi=1&jsapiver=v1&newsession=1&corrid=585438e0-6afc-4938-8831-11bc73244cd8&usid=585438e0-6afc-4938-8831-11bc73244cd8&sftc=1&cac=1&mtf=1&sfp=1&wdredirectionreason=Unified_SingleFlush&rct=Normal&ctp=LeastProtected#_ftn2) was tested and rated as B2, sufficient for those with conversational English abilities.

The survey was translated into Spanish, French, Swahili/Sheng, Hindi, Urdu, Italian, Bangla, Arabic, and Chinese through a combination of an initial automated translation (using Google Translate (26)) followed by proof reading and correction by native language speakers in each language. Data were collected from August 17^th^ to October 10^th^ 2021 on respondents’ own devices.

**Data Storage**

All responses were stored on the Typeform.com servers (hosted in a Virtual Private Cloud by Amazon Web Services) and processed and analysed on password-protected encrypted devices. All data collection tools, anonymised raw data, and data analysis code are available on the LSHTM Data Compass Site (14) mirrored on Figshare (27). The dataset has been assigned a unique DataCite DOI (28). Confidentiality of survey responses was maintained throughout; no personally identifiable data was collected. Age in years was collected, and the location was recorded at the city level. In addition, no IP address or other locator/identifiers (like cookies) were captured.

[^[1]^](https://euc-word-edit.officeapps.live.com/we/wordeditorframe.aspx?new=1&ui=en%2DUS&rs=en%2DUS&wopisrc=https%3A%2F%2Flshtm.sharepoint.com%2Fsites%2FCCC_Group%2F_vti_bin%2Fwopi.ashx%2Ffiles%2Fabe8096bc52942738c40e63e20c428c5&wdenableroaming=1&mscc=1&hid=E08E9CA0-2057-6000-48B7-7F9B4CAE206B&wdorigin=Other&jsapi=1&jsapiver=v1&newsession=1&corrid=585438e0-6afc-4938-8831-11bc73244cd8&usid=585438e0-6afc-4938-8831-11bc73244cd8&sftc=1&cac=1&mtf=1&sfp=1&wdredirectionreason=Unified_SingleFlush&rct=Normal&ctp=LeastProtected#_ftnref1) [Flesh Reading Ease Score](https://readable.com/readability/flesch-reading-ease-flesch-kincaid-grade-level/#:~:text=The%20Flesch%20Reading%20Ease%20gives,the%201940s%20by%20Rudolf%20Flesch.)

[^[2]^](https://euc-word-edit.officeapps.live.com/we/wordeditorframe.aspx?new=1&ui=en%2DUS&rs=en%2DUS&wopisrc=https%3A%2F%2Flshtm.sharepoint.com%2Fsites%2FCCC_Group%2F_vti_bin%2Fwopi.ashx%2Ffiles%2Fabe8096bc52942738c40e63e20c428c5&wdenableroaming=1&mscc=1&hid=E08E9CA0-2057-6000-48B7-7F9B4CAE206B&wdorigin=Other&jsapi=1&jsapiver=v1&newsession=1&corrid=585438e0-6afc-4938-8831-11bc73244cd8&usid=585438e0-6afc-4938-8831-11bc73244cd8&sftc=1&cac=1&mtf=1&sfp=1&wdredirectionreason=Unified_SingleFlush&rct=Normal&ctp=LeastProtected#_ftnref2) [CEFR Level](https://readable.com/readability/cefr-test/)
