# Supplementary material for: Let’s just ask them. Perspectives on urban dwelling and air quality: A cross-sectional survey of 3,222 children, young people and parents
Source: PLOS Glob Public Health. 2023 Apr 13;3(4):e0000963. doi: 10.1371/journal.pgph.0000963 (PMC10101632; doi:10.1371/journal.pgph.0000963)
Supplement: S3 Appendix — (DOCX) [file pgph.0000963.s003.docx]

# **S3 Appendix: Survey instrument**

| *Description or Typeform survey text* | *Answer options* |
| --- | --- |
| *“How old are you?”* | ***Open text*** |
| *“Are you or your partner currently pregnant?”* | ***Single choice:***  *Pregnant*  *Not pregnant* |
| *“Where do you live?”* | ***Single choice:***  *Bubaneswar (India)*  *Dar es Salaam (Tanzania)*  *Dhaka (Bangladesh)*  *Freetown (Sierra Leone)*  *Glasgow (Scotland)*  *Jaipur (India)*  *Lahore (Pakistan)*  *London (England)*  *Los Angeles (USA)*  *Milan (Italy)*  *Quito (Ecuador)*  *Tamale (Ghana)* |
| *“What are the BEST things about living in your city/town?”* | ***Multiple selection, up to three:***   *The people*  *Places to play*  *Being close to school or work*  *There are many things to do*  *The shops and restaurants*  *It's easy to get around*  *Healthcare*  *Being close to my family*  *The climate and environment*  *Work opportunities for my family*  *Access to green space, like parks*  *Other* |
| *“Now, what are the WORST things about living in your city/town?”* | ***Multiple selection, up to three:***  *It doesn't always feel safe*  *The people; it's not friendly*  *The traffic/congestion*  *The noise*  *Not enough places to play*  *Not enough places to meet friends*  *Shortage of work opportunities for my family*  *It's hard to get around*  *Not easy enough to get healthcare*  *The pollution*  *It's too crowded*  *Not enough green space like parks*  *Other* |
| *“Do you have any comments to add (about your city in general)?”* | ***Open text*** |
| *“We are interested in what you think about the air quality where you live”* | ***Single choice:***  *Numeric 0-10* |
| *“What do you think are the main sources of AP where you live”* | ***Multiple selection, unlimited:***  *Factories*  *Burning of rubbish*  *Motor transport (cars, buses, lorries)*  *Construction/building work*  *Pollution blown into the city (from outside)*  *Household cooking (cooking fires/stoves)*  *Household heating (boilers, wood fires etc)*  *Agriculture/farming*  *Other* |
| *“Do you have any comments to add (about the air quality in your city)?”* | ***Open text*** |
| *“In general, is your city becoming a nicer place to live, or a less nice place to live?”* | ***Single choice:***  *Becoming a nicer place to live*  *Staying the same*  *Becoming a worse place to live* |
| *“Why do you say that?”*  *Qualitative responses to the nicer_city question* | ***Open text*** |
| *“If you were the mayor of your town/city, how would you…Improve the city generally, especially for children and young people?”* | ***Open text*** |
| *“And, how would you improve air quality where you live?”* | ***Open text*** |
| *“Do you have any more comments about any of these issues?”* | ***Open text*** |
