# Supplementary material for: Let’s just ask them. Perspectives on urban dwelling and air quality: A cross-sectional survey of 3,222 children, young people and parents
Source: PLOS Glob Public Health. 2023 Apr 13;3(4):e0000963. doi: 10.1371/journal.pgph.0000963 (PMC10101632; doi:10.1371/journal.pgph.0000963)
Supplement: S4 Appendix — (DOCX) [file pgph.0000963.s004.docx]

# **S4 Appendix: Language, consent, and eligibility questions**

| *Variable Name* | *Description or Typeform survey text* | *Answer options* |
| --- | --- | --- |
| *Lang* | *Hello presented in each language* | ***Single Choice:***  *Hello*  *Hola*  *Bonjour*  *Niaje*  *नमस्ते*  *ہیلو*  *Ciao*  *হ্যালো*  *أهلا*  *你好* |
| *Eligibility 1* | *Before we start, we need to tell you about this research so you can decide if you want to take part.*  ***Who are we?*** *We are a group of researchers from London School of Hygiene and Tropical Medicine who are asking children, young people and parents from all over the world to tell us what they think of their cities. We think children’s voices and opinions are not included enough, and want to try to address this.*  ***Why are you seeing this survey?*** *We paid for posts to appear in Facebook/Instagram feeds of people like you who live in your city. (This is based on the information that Facebook/Instagram holds about you, not any information we have – we do not have, or have access to, any of your personal information at all).*  ***How long is it?*** *We have 10 questions for you, that will take you less than 5 minutes to answer.*  ***How can I contact you if I have questions?*** *You can email us (the study leads Prof Alan Dangour and Dr Rob Hughes) at* [*ccc@lshtm.ac.uk*](mailto:ccc@lshtm.ac.uk) *if you have any questions about this survey.*  ***To take part you must be:***  *Aged between 13-25 years old AND live in a town/city, OR*  *A parent of someone under 13 years old AND live in a town/city, OR*  *Aged over 18, you or your partner are expecting a baby AND live in a town/city*  ***Select the option that best describes you:*** | ***Single choice:***  *Aged between 13-25 years old*  *A parent of someone under 13 years old*  *Aged over 18, you or your partner are expecting a baby*  *None of these describe me* |
| *Consent 1* | *Participation is completely voluntary; you don't have to take part and you can stop at any time* | ***Single choice:***  *OK, I want to complete the survey*  *No, I don't want to complete the survey* |
| *Consent 2* | *Your answers are anonymous – that means nobody will know who you are and nobody will be able to connect your answers back to you (including the researchers)* | ***Single choice:***  *OK, I want to complete the survey*  *No, I don't want to complete the survey* |
| *Consent 3* | *We will write research reports, blogs, and social media posts based on what we learn from all survey responses combined together* | ***Single choice:***  *OK, I want to complete the survey*  *No, I don't want to complete the survey* |
| *Consent 4* | *Storing and sharing data: We will keep your answers securely on our computers, and we might also share them with other scientists in the future too (we will never have or share any information that could identify you individually)* | ***Single choice:***  *OK, I want to complete the survey*  *No, I don't want to complete the survey* |
| *Consent 5* | *You will not get any reward or prize for taking part*  *(To show our thanks, you will receive a certificate of participation at the end that you can print out.)* | ***Single choice:***  *OK, I want to complete the survey*  *No, I don't want to complete the survey* |
| *Question 1* | *Do you have any questions?* | ***Single choice:***  *Yes - Take me to the FAQs*  *No - START survey* |
| *FAQs* | *FAQs*  ***Why are you doing this work?***  *We are really keen to learn what children, young people and parents (and people who are soon to become parents) think of their city. We think children’s voices and opinions are not included enough, and want to try to address this.*  ***2. How did you select me?***  *We want to hear from people who live in your city who are children/young people, parents of children or expecting a baby soon. We paid for posts to appear in Facebook/Instagram feeds of people like you who live in your city. (This is based on the information that Facebook/Instagram holds about you, not any information we have – we do not have, or have access to, any personal information at all).*  ***3. Why do I not get anything in return for completing the survey?***  *Firstly, this is a very short survey – it will probably only take you up to 6-7 minutes to complete it. Also, sending you a thank you for your time would require collecting personal information about you, like your phone number or email address; we don’t want to collect and store that information, so thought it would be simpler to simply THANK YOU for your time if you do decide to complete the survey. (There is a certificate you can print at the end if you'd like to).*  ***4. Who are you?***  *We are researchers at the London School of Hygiene and Tropical Medicine (lead researchers Prof Alan Dangour and Dr Rob Hughes), in the United Kingdom. This work is funded by the Botnar Foundation (which is based in Switzerland).*  ***5. How will you keep my answers private?***  *Firstly, we are deliberately not collecting any personal information which could identify you from the many thousands of responses we’re hoping to collect. Secondly, all the responses are kept safely on a highly secure computer.* | ***Single choice:***  *Yes - get in touch*  *No – START survey* |
