# Supplementary material for: Let’s just ask them. Perspectives on urban dwelling and air quality: A cross-sectional survey of 3,222 children, young people and parents
Source: PLOS Glob Public Health. 2023 Apr 13;3(4):e0000963. doi: 10.1371/journal.pgph.0000963 (PMC10101632; doi:10.1371/journal.pgph.0000963)
Supplement: S5 Appendix — (DOCX) [file pgph.0000963.s005.docx]

# **S5 Appendix: Survey completions by country**

| Country | Frequency | Percent |
| --- | --- | --- |
| Bangladesh | 866 | 26.88 |
| India | 337 | 10.46 |
| Pakistan | 325 | 10.09 |
| Ghana | 301 | 9.34 |
| Ecuador | 288 | 8.94 |
| Zimbabwe | 276 | 8.57 |
| Kenya | 174 | 5.4 |
| United Kingdom | 170 | 5.28 |
| Sierra Leone | 160 | 4.97 |
| Tanzania | 96 | 2.98 |
| Mexico | 90 | 2.79 |
| Italy | 71 | 2.2 |
| Philippines | 57 | 1.77 |
| United States of America | 11 | 0.34 |
|  |  |  |
|  |  |  |
| Total | 3,222 | 100 |
