# Supplementary material for: Let’s just ask them. Perspectives on urban dwelling and air quality: A cross-sectional survey of 3,222 children, young people and parents
Source: PLOS Glob Public Health. 2023 Apr 13;3(4):e0000963. doi: 10.1371/journal.pgph.0000963 (PMC10101632; doi:10.1371/journal.pgph.0000963)
Supplement: S6 Appendix — (DOCX) [file pgph.0000963.s006.docx]

# **S6 Appendix: Respondents by self-reported town**

| **Town** | **Frequency** | **Percent** |  | **Town** | **Frequency** | **Percent** |
| --- | --- | --- | --- | --- | --- | --- |
| Dhaka † | 858 | 26.63 |  | Chitral | 1 | 0.03 |
| Lahore † | 315 | 9.78 |  | Chitungwiza | 1 | 0.03 |
| Tamale † | 300 | 9.31 |  | Cobham | 1 | 0.03 |
| Quito † | 288 | 8.94 |  | Cover | 1 | 0.03 |
| Harare † | 271 | 8.41 |  | Echague | 1 | 0.03 |
| Jaipur † | 225 | 6.98 |  | Entebbe** | 1 | 0.03 |
| Nairobi † | 169 | 5.25 |  | Firozabad | 1 | 0.03 |
| Freetown † | 159 | 4.93 |  | Groningen | 1 | 0.03 |
| Glasgow † | 113 | 3.51 |  | Haroonabad | 1 | 0.03 |
| Bhubaneswar † | 104 | 3.23 |  | Jhang | 1 | 0.03 |
| Dar es Salaam † | 96 | 2.98 |  | Kajiado | 1 | 0.03 |
| Mexico City † | 89 | 2.76 |  | Khulna | 1 | 0.03 |
| Milan † | 69 | 2.14 |  | Kuchaman | 1 | 0.03 |
| London † | 56 | 1.74 |  | Mardan | 1 | 0.03 |
| Quezon City † | 36 | 1.12 |  | Marikina | 1 | 0.03 |
| Manila | 13 | 0.4 |  | Masvingo | 1 | 0.03 |
| Los Angeles † | 11 | 0.34 |  | Monza | 1 | 0.03 |
| missing* | 4 | 0.12 |  | Muranga | 1 | 0.03 |
| Narayanganj | 3 | 0.09 |  | Mymensingh | 1 | 0.03 |
| Churu | 2 | 0.06 |  | Narowal | 1 | 0.03 |
| Sialkot | 2 | 0.06 |  | New York | 1 | 0.03 |
| Victoria Falls | 2 | 0.06 |  | Okara | 1 | 0.03 |
| Alwar | 1 | 0.03 |  | Rourkela | 1 | 0.03 |
| Antipolo | 1 | 0.03 |  | San Jose Del Monte | 1 | 0.03 |
| Atrai | 1 | 0.03 |  | San Juan | 1 | 0.03 |
| Bahawalnagar | 1 | 0.03 |  | Sargodha | 1 | 0.03 |
| Baripada | 1 | 0.03 |  | Sirsa | 1 | 0.03 |
| Bomet | 1 | 0.03 |  | Valenzuela | 1 | 0.03 |
| Busia | 1 | 0.03 |  | Yeji | 1 | 0.03 |
| Caloocan | 1 | 0.03 |  | **Total** | **3,222** | **100** |

*people who did not explicitly state a town and could not impute from paid media advert campaign meta data

**individual reported living in Entebbe Uganda, but paid media advert campaign meta data identified them as living in Nairobi Kenya. The response was clustered with Nairobi responses.

† City included in the social media campaign advertisements
