# Supplementary material for: Let’s just ask them. Perspectives on urban dwelling and air quality: A cross-sectional survey of 3,222 children, young people and parents
Source: PLOS Glob Public Health. 2023 Apr 13;3(4):e0000963. doi: 10.1371/journal.pgph.0000963 (PMC10101632; doi:10.1371/journal.pgph.0000963)
Supplement: S7 Appendix — (DOCX) [file pgph.0000963.s007.docx]

# **S7 Appendix: Distribution of respondents (n=3,222) and focal cities (n=16) by pm2.5 and income indicators**

| PM_2.5_ quartile and represented cities | n(%) of  n=3,222 sample | Mean (sd)  pm2.5 (ug/m3) | World Bank  income group | Gross domestic  product per  capita (USD) |
| --- | --- | --- | --- | --- |
| **Quartile 1** | **113 (4%)** | **8.00 (0)** |  |  |
| Glasgow | 113 | 8.00 | High | 34,358 |
| **Quartile 2** | **68 (2%)** | **12.00 (0)** |  |  |
| London | 57 | 12.00 | High | 58,827 |
| Los Angeles | 11 | 12.00 | High | 57,577 |
| **Quartile 3** | **1,045 (32%)** | **19.86 (2.21)** |  |  |
| Quito | 288 | 18.0 | Upper-middle | 6,368 |
| Nairobi | 174 | 17.00 | Lower-middle | 6,344 |
| Quezon City | 57 | 18.00 | Lower-middle | 8,482 |
| Freetown | 160 | 21.63 | Low | 1,079 |
| Mexico City | 90 | 22.00 | Upper-middle | 22,587 |
| Harare | 276 | 22.25 | Lower-middle | 1,386 |
| **Quartile 4** | **1,996 (62%)** | **63.03 (18.88)** |  |  |
| Milan | 71 | 27.00 | High | 51,768 |
| Dar es Salaam | 96 | 29.08 | Lower-middle | 5,699 |
| Tamale | 301 | 55.00 | Lower-middle | 5,150 |
| Dhaka | 866 | 57.00 | Lower-middle | 7,712 |
| Lahore | 325 | 68.00 | Lower-middle | 3,144 |
| Bhubaneswar | 106 | 83.09 | Lower-middle | 1,300 |
| Jaipur | 231 | 105.00 | Lower-middle | 1,500 |
