# Supplementary material for: Let’s just ask them. Perspectives on urban dwelling and air quality: A cross-sectional survey of 3,222 children, young people and parents
Source: PLOS Glob Public Health. 2023 Apr 13;3(4):e0000963. doi: 10.1371/journal.pgph.0000963 (PMC10101632; doi:10.1371/journal.pgph.0000963)
Supplement: S8 Appendix — (DOCX) [file pgph.0000963.s008.docx]

# **S8 Appendix: Demographics for full sample and each subpopulation**

|  | Total  n (%) | Age  Mean (SD) | Parent of a Child  Aged <13  n(%) | Young People  Aged 13-25  n (%) | Expectant Parents  n (%) |
| --- | --- | --- | --- | --- | --- |
| ‘Best’ | 2,939 (100%) | 24 (8.26) | 496 (17%) | 2,187 (74%) | 267 (9%) |
| ‘Worst’ | 3,051 (100%) | 24 (8.50) | 508 (17%) | 2,266 (74%) | 277 (9%) |
| ‘Pollution’ | 3,056 (100%) | 24 (8.50) | 509 (17%) | 2,270 (74%) | 277 (9%) |
| ‘Air Quality’ | 3,054 (100%) | 24 (8.50) | 509 (17%) | 2,267 (74%) | 278 (9%) |
| ‘Nicer City’ | 3,061 (100%) | 24 (8.49) | 509 (17%) | 2,273 (74%) | 279 (9%) |
| **Full Sample** | **3,222 (100%)** | **24 (8.26)** | **509 (16%)** | **2,430 (75%)** | **283 (9%)** |
