# Supplementary material for: Let’s just ask them. Perspectives on urban dwelling and air quality: A cross-sectional survey of 3,222 children, young people and parents
Source: PLOS Glob Public Health. 2023 Apr 13;3(4):e0000963. doi: 10.1371/journal.pgph.0000963 (PMC10101632; doi:10.1371/journal.pgph.0000963)
Supplement: S9 Appendix — (DOCX) [file pgph.0000963.s009.docx]

# **S9 Appendix: Distribution of respondent demographics in total (n=3,222) and by focal city**

|  |  |  |  |  |  |  |  |  |  |  |
| --- | --- | --- | --- | --- | --- | --- | --- | --- | --- | --- |
|  |  | Target Population |  | **Age Buckets** | | | | | | |
|  | Total | Parent or expecting | Young person | Total | Unknown | 13-16 | 17-19 | 20-25 | | 25+ |
|  | n  % | n  % | n  % | n  % | n  % | n  % | n  % | n  % | n  % | |
| **Bhubaneswar** | 106 | 26 | 80 | 106 | 0 | 7 | 19 | 58 | 22 | |
|  | 100% | 25% | 75% | 100% | 0% | 7% | 18% | 55% | 21% | |
| **Dar es Salaam** | 96 | 41 | 55 | 96 | 1 | 3 | 8 | 51 | 33 | |
|  | 100% | 43% | 57% | 100% | 1% | 3% | 8% | 53% | 34% | |
| **Dhaka** | 866 | 127 | 739 | 866 | 8 | 108 | 235 | 415 | 100 | |
|  | 100% | 15% | 85% | 100% | 1% | 12% | 27% | 48% | 12% | |
| **Freetown** | 160 | 41 | 119 | 160 | 0 | 9 | 26 | 89 | 36 | |
|  | 100% | 26% | 74% | 100% | 0% | 6% | 16% | 56% | 23% | |
| **Glasgow** | 113 | 14 | 99 | 113 | 0 | 24 | 29 | 47 | 13 | |
|  | 100% | 12% | 88% | 100% | 0% | 21% | 26% | 42% | 12% | |
| **Harare** | 276 | 79 | 197 | 276 | 1 | 16 | 31 | 162 | 66 | |
|  | 100% | 29% | 71% | 100% | 0% | 6% | 11% | 59% | 24% | |
| **Jaipur** | 231 | 63 | 168 | 231 | 0 | 7 | 38 | 148 | 38 | |
|  | 100% | 27% | 73% | 100% | 0% | 3% | 16% | 64% | 16% | |
| **Lahore** | 325 | 163 | 162 | 325 | 0 | 29 | 71 | 104 | 121 | |
|  | 100% | 50% | 50% | 100% | 0% | 9% | 22% | 32% | 37% | |
| **London** | 57 | 8 | 49 | 57 | 0 | 22 | 11 | 20 | 4 | |
|  | 100% | 14% | 86% | 100% | 0% | 39% | 19% | 35% | 7% | |
| **Los Angeles** | 11 | 2 | 9 | 11 | 0 | 3 | 2 | 4 | 2 | |
|  | 100% | 18% | 82% | 100% | 0% | 27% | 18% | 36% | 18% | |
| **Mexico City** | 90 | 17 | 73 | 90 | 0 | 22 | 28 | 23 | 17 | |
|  | 100% | 19% | 81% | 100% | 0% | 24% | 31% | 26% | 19% | |
| **Milan** | 71 | 12 | 59 | 71 | 0 | 8 | 14 | 38 | 11 | |
|  | 100% | 17% | 83% | 100% | 0% | 11% | 20% | 54% | 15% | |
| **Nairobi** | 174 | 73 | 101 | 174 | 0 | 4 | 15 | 98 | 57 | |
|  | 100% | 42% | 58% | 100% | 0% | 2% | 9% | 56% | 33% | |
| **Quezon City** | 57 | 9 | 48 | 57 | 0 | 9 | 22 | 17 | 9 | |
|  | 100% | 16% | 84% | 100% | 0% | 16% | 39% | 30% | 16% | |
| **Quito** | 288 | 60 | 228 | 288 | 1 | 48 | 79 | 110 | 50 | |
|  | 100% | 21% | 79% | 100% | 0% | 17% | 27% | 38% | 17% | |
| **Tamale** | 301 | 57 | 244 | 301 | 3 | 28 | 217 | 53 | 0 | |
|  | 100% | 19% | 81% | 100% | 1% | 9% | 72% | 18% | 0% | |
| **All cities** | 3222 | 792 | 2430 | 3211 | 322 | 656 | 1601 | 632 | 0 | |
|  | 100% | 25% | 75% | 100% | 10% | 20% | 50% | 20% | 0% | |
