# Supplementary material for: Let’s just ask them. Perspectives on urban dwelling and air quality: A cross-sectional survey of 3,222 children, young people and parents
Source: PLOS Glob Public Health. 2023 Apr 13;3(4):e0000963. doi: 10.1371/journal.pgph.0000963 (PMC10101632; doi:10.1371/journal.pgph.0000963)
Supplement: S10 Appendix — (DOCX) [file pgph.0000963.s010.docx]

# **S10 Appendix: Percentage of total n respondents that reported each item within the top 3 ‘best’ aspects of their cities, stratified by PM_2.5_ quartile, age bucket, and respondent group**

|  |  |  |  |  |  |  |  |  |  |  |  |  |  |
| --- | --- | --- | --- | --- | --- | --- | --- | --- | --- | --- | --- | --- | --- |
|  | Total n (100%*) | Being close to my family | There are many things to do | Being close to school or work | The shops and restaurants | Work opportunities for my family | The People | It's easy to get around | The climate and environment | Access to green spaces, like parks | Healthcare | Places to Play | Other |
| **Full sample** | 2,939 (100%) | 30% | 27% | 27% | 24% | 22% | 22% | 21% | 18% | 14% | 13% | 4% | 3% |
| **PM_2.5_ Quartile** | |  |  |  |  |  |  |  |  |  |  |  |  |
| 1 | 109 (100%) | 21% | 33% | 39% | 45% | 10% | 36% | 25% | 6% | 33% | 20% | 4% | 4% |
| 2 | 64 (100%) | 13% | 45% | 25% | 39% | 20% | 16% | 30% | 6% | 23% | 17% | 3% | 0% |
| 3 | 863 (100%) | 32% | 24% | 22% | 16% | 19% | 24% | 17% | 32% | 14% | 9% | 3% | 2% |
| 4 | 1,903 (100%) | 29% | 27% | 28% | 26% | 24% | 20% | 22% | 13% | 12% | 14% | 4% | 1% |
| **Age bucket** |  |  |  |  |  |  |  |  |  |  |  |  |  |
| Unknown | 9 (100%) | 44% | 11% | 0% | 22% | 0% | 44% | 0% | 33% | 11% | 0% | 11% | 0% |
| 13-16 | 303 (100%) | 24% | 30% | 28% | 33% | 17% | 19% | 18% | 16% | 19% | 14% | 5% | 2% |
| 17-19 | 603 (100%) | 28% | 29% | 31% | 27% | 19% | 20% | 18% | 16% | 12% | 13% | 5% | 1% |
| 20-25 | 1,411 (100%) | 28% | 27% | 25% | 23% | 22% | 22% | 23% | 18% | 14% | 12% | 3% | 1% |
| 25+ | 613 (100%) | 37% | 23% | 25% | 21% | 28% | 23% | 19% | 19% | 14% | 15% | 2% | 1% |
| **Respondent group** |  |  |  |  |  |  |  |  |  |  |  |  |  |
| Parent or expectant | 762 (100%) | 34% | 23% | 25% | 20% | 27% | 21% | 19% | 19% | 15% | 14% | 4% | 1% |
| Young person | 2,177 (100%) | 28% | 28% | 27% | 25% | 21% | 22% | 21% | 17% | 14% | 13% | 4% | 1% |
|  |  |  |  |  |  |  |  |  |  |  |  |  |  |
| *Stratified percentages add up to over 100% | | | | | | | | | | |  |  |  |
