# Supplementary material for: Let’s just ask them. Perspectives on urban dwelling and air quality: A cross-sectional survey of 3,222 children, young people and parents
Source: PLOS Glob Public Health. 2023 Apr 13;3(4):e0000963. doi: 10.1371/journal.pgph.0000963 (PMC10101632; doi:10.1371/journal.pgph.0000963)
Supplement: S11 Appendix — (DOCX) [file pgph.0000963.s011.docx]

# **S11 Appendix: The percentage of total n respondents that reported each item within the top 3 ‘worst’ aspects of their cities, stratified by PM_2.5_ quartile, age bucket, and respondent group**

|  |  |  |  |  |  |  |  |  |  |  |  |  |  |  |
| --- | --- | --- | --- | --- | --- | --- | --- | --- | --- | --- | --- | --- | --- | --- |
|  | Total n (100%*) | The traffic/  congestion | The pollution | Shortage of work opportunities for my family | It's too crowded | It doesn't always feel safe | The noise | Not enough green spaces like parks | Not easy enough to get healthcare | Not enough places to play | It's hard to get around | Not enough places to meet friends | The people; it's not friendly | Other |
| **Full sample** | 3,051 (100%) | 49% | 39% | 23% | 23% | 20% | 16% | 14% | 14% | 11% | 7% | 5% | 5% | 5% |
| **PM_2.5_ Quartile** |  |  |  |  |  |  |  |  |  |  |  |  |  |  |
| 1 | 113 (100%) | 41% | 34% | 12% | 7% | 54% | 14% | 7% | 4% | 7% | 20% | 14% | 10% | 10% |
| 2 | 67 (100%) | 48% | 42% | 1% | 33% | 45% | 16% | 9% | 4% | 4% | 7% | 7% | 9% | 3% |
| 3 | 898 (100%) | 51% | 31% | 36% | 20% | 27% | 11% | 11% | 23% | 4% | 7% | 5% | 4% | 5% |
| 4 | 1973 (100%) | 48% | 42% | 18% | 24% | 14% | 18% | 16% | 10% | 14% | 6% | 4% | 5% | 4% |
| **Age bucket** |  |  |  |  |  |  |  |  |  |  |  |  |  |  |
| Unknown | 11 (100%) | 27% | 36% | 36% | 9% | 27% | 18% | 9% | 0% | 27% | 0% | 9% | 0% | 9% |
| 13-16 | 321 (100%) | 45% | 46% | 12% | 19% | 36% | 17% | 12% | 8% | 14% | 5% | 7% | 5% | 2% |
| 17-19 | 621 (100%) | 46% | 43% | 17% | 22% | 25% | 19% | 14% | 12% | 12% | 6% | 5% | 7% | 4% |
| 20-25 | 1468 (100%) | 48% | 37% | 27% | 23% | 15% | 15% | 14% | 15% | 9% | 7% | 5% | 4% | 6% |
| 25+ | 630 (100%) | 55% | 35% | 24% | 24% | 16% | 14% | 15% | 16% | 12% | 9% | 3% | 4% | 4% |
| **Respondent group** |  |  |  |  |  |  |  |  |  |  |  |  |  |  |
| Parent or expectant | 785 (100%) | 54% | 35% | 23% | 24% | 16% | 15% | 14% | 15% | 11% | 9% | 4% | 5% | 3% |
| Young person | 2,266 (100%) | 47% | 40% | 23% | 22% | 21% | 16% | 14% | 13% | 11% | 6% | 6% | 5% | 5% |
| *Stratified percentages add up to over 100% | | | | | | | | | | |  |  |  |  |
