# Supplementary material for: Let’s just ask them. Perspectives on urban dwelling and air quality: A cross-sectional survey of 3,222 children, young people and parents
Source: PLOS Glob Public Health. 2023 Apr 13;3(4):e0000963. doi: 10.1371/journal.pgph.0000963 (PMC10101632; doi:10.1371/journal.pgph.0000963)
Supplement: S12 Appendix — (DOCX) [file pgph.0000963.s012.docx]

# **S12 Appendix: The percentage of total n respondents that reported better, worse, or no changes to their cities, stratified by PM_2.5_ quartile, age bucket, and respondent group**

|  | Total n (%) | Becoming a nicer  place to live | Staying  the same | Becoming a worse  place to live |
| --- | --- | --- | --- | --- |
| **Full sample** | 2,993 (100%) | 43% | 23% | 34% |
| **PM_2.5_ quartile** |  |  |  |  |
| 1 | 113 (100%) | 43% | 37% | 19% |
| 2 | 67 (100%) | 39% | 28% | 33% |
| 3 | 898 (100%) | 31% | 30% | 39% |
| 4 | 1,915 (100%) | 48% | 19% | 33% |
| **Age bucket** |  |  |  |  |
| Unknown | 11 (100%) | 18% | 45% | 36% |
| 13-16 | 312 (100%) | 45% | 29% | 26% |
| 17-19 | 611 (100%) | 43% | 26% | 31% |
| 20-25 | 1,437 (100%) | 44% | 22% | 34% |
| 25+ | 622 (100%) | 38% | 20% | 42% |
| **Respondent group** |  |  |  |  |
| Parent or expectant | 778 (100%) | 39% | 22% | 40% |
| Young person | 2,215 (100%) | 44% | 24% | 32% |
|  |  |  |  |  |
