# Supplementary material for: Let’s just ask them. Perspectives on urban dwelling and air quality: A cross-sectional survey of 3,222 children, young people and parents
Source: PLOS Glob Public Health. 2023 Apr 13;3(4):e0000963. doi: 10.1371/journal.pgph.0000963 (PMC10101632; doi:10.1371/journal.pgph.0000963)
Supplement: S13 Appendix — (DOCX) [file pgph.0000963.s013.docx]

# **S13 Appendix: Mean air quality (scale 0-10) reported by n=3,054 respondents, stratified by PM_2.5_ quartile**

|  | Total n (%) | Mean Air Quality  (Likert Scale 0-10) | SD |
| --- | --- | --- | --- |
| **Full sample** | 3,054 (100%) | 5.07 | 2.86 |
| **PM_2.5_ quartile** |  |  |  |
| 1 | 113 (4%) | 6.01 | 2.19 |
| 2 | 67 (2%) | 5.31 | 2.42 |
| 3 | 897 (29%) | 5.73 | 2.64 |
| 4 | 1,977 (65%) | 4.71 | 2.94 |
| **Age bucket** |  |  |  |
| Unknown | 11 (0.4%) | 4.73 | 3.07 |
| 13-16 | 320 (10%) | 5.25 | 2.81 |
| 17-19 | 622 (20%) | 4.97 | 2.88 |
| 20-25 | 1,469 (48%) | 5.11 | 2.89 |
| 25+ | 632 (21%) | 4.99 | 2.79 |
| **Respondent group** |  |  |  |
| Parent or expectant | 787 (26%) | 5.04 | 2.76 |
| Young person | 2,267 (74%) | 5.08 | 2.9 |
