# Supplementary material for: Let’s just ask them. Perspectives on urban dwelling and air quality: A cross-sectional survey of 3,222 children, young people and parents
Source: PLOS Glob Public Health. 2023 Apr 13;3(4):e0000963. doi: 10.1371/journal.pgph.0000963 (PMC10101632; doi:10.1371/journal.pgph.0000963)
Supplement: S14 Appendix — (DOCX) [file pgph.0000963.s014.docx]

# **S14 Appendix: The percentage of total n respondents who reported each major source of AP in their city, stratified by PM_2.5_ quartile, age bucket, and respondent group**

|  |  |  |  |  |  |  |  |  |  |  |
| --- | --- | --- | --- | --- | --- | --- | --- | --- | --- | --- |
|  | Total n (100%*) | Motor transport (cars, buses, lorries) | Factories | Burning of rubbish | Construction/building work | Pollution blown into the city (from outside) | Household cooking (cooking fires/stoves) | Household heating (boilers, wood fires etc) | Agriculture/farming | Other |
| **Full Sample** | 3,056 (100%) | 32% | 18% | 17% | 14% | 8% | 5% | 5% | 2% | 1% |
| **PM_2.5_ Quartile** |  |  |  |  |  |  |  |  |  |  |
| 1 | 113 (100%) | 90% | 35% | 18% | 44% | 13% | 20% | 12% | 3% | 4% |
| 2 | 67 (100%) | 81% | 28% | 12% | 40% | 15% | 16% | 7% | 4% | 3% |
| 3 | 898 (100%) | 77% | 39% | 49% | 21% | 16% | 10% | 13% | 4% | 2% |
| 4 | 1,978 (100%) | 75% | 44% | 37% | 36% | 20% | 11% | 10% | 4% | 2% |
| **Age bucket** |  |  |  |  |  |  |  |  |  |  |
| Unknown | 11 (100%) | 64% | 45% | 55% | 18% | 9% | 0% | 9% | 9% | 9% |
| 13-16 | 321 (100%) | 79% | 38% | 36% | 33% | 18% | 12% | 6% | 2% | 2% |
| 17-19 | 623 (100%) | 74% | 50% | 39% | 34% | 19% | 11% | 9% | 4% | 1% |
| 20-25 | 1,470 (100%) | 74% | 42% | 42% | 32% | 19% | 13% | 12% | 4% | 2% |
| 25+ | 631 (100%) | 81% | 37% | 36% | 29% | 17% | 8% | 12% | 3% | 2% |
| **Respondent group** |  |  |  |  |  |  |  |  |  |  |
| Parent or expectant | 786 (100%) | 79% | 38% | 36% | 27% | 18% | 11% | 8% | 4% | 2% |
| Young person | 2,270 (100%) | 75% | 43% | 41% | 33% | 19% | 10% | 12% | 4% | 2% |
| *Stratified percentages add up to over 100% | | | | | | | | | | |
