# Supplementary material for: Let’s just ask them. Perspectives on urban dwelling and air quality: A cross-sectional survey of 3,222 children, young people and parents
Source: PLOS Glob Public Health. 2023 Apr 13;3(4):e0000963. doi: 10.1371/journal.pgph.0000963 (PMC10101632; doi:10.1371/journal.pgph.0000963)
Supplement: S15 Appendix — (DOCX) [file pgph.0000963.s015.docx]

# **S15 Appendix: Illustrative quotes of youth's specific ideas and asks for their cities, by subtheme**

**Concerns about inequality**

“Easy Living For Rich People, Hard for Poor or Middle Class Family”

**Corruption and bad governance**

“Corruption is the root of all these problems. None can solve these issues without solving corruption”

**Young people being absent from decision making**

“Engage the young people to be in the front"

**A lack of consciousness for the environment**

“People should be trained to be vigilant about environment problems”
