# Supplementary material for: Let’s just ask them. Perspectives on urban dwelling and air quality: A cross-sectional survey of 3,222 children, young people and parents
Source: PLOS Glob Public Health. 2023 Apr 13;3(4):e0000963. doi: 10.1371/journal.pgph.0000963 (PMC10101632; doi:10.1371/journal.pgph.0000963)
Supplement: S16 Appendix — (DOCX) [file pgph.0000963.s016.docx]

# **S16 Appendix: Illustrative quotes of structural barriers to change, by subtheme**

**City Design and Space**

*“There is no other way than planting more trees”*

*“Building playground, parks, and offering clean and safe environment”*

**Urban Mobility**

*“Creating a massive, comfortable, fast and efficient transportation system”*

*“Make more roads into pedestrian areas and build more bike lanes.”*

**Health**

*“Healthcare must be free of cost especially primary health care”*

*“Health care system is a mess, if you don’t have money you’ll die because they won’t attend to you”*

**Education**

*“…improve the education system by making it more fun to learn…”*

*“For young: quality education, proper knowledge of how contemporary world and near future world works.”*

**Skills and jobs**

*“We need job opportunities”*

*“I would firstly improve job opportunities and salary's cause one can only establish a stable life when they have a house as an asset and not liability and have a constant source of income which they use to establish or live an average life without the fear of poverty.”*

**Other basic services**

*“Improve housing, food and water supply”*

*“basic services such as clean water and or electricity are not consistent or available for a lot of people. There is no refuse collection and raw sewerage flowing in many areas.”*
