# Supplementary material for: Let’s just ask them. Perspectives on urban dwelling and air quality: A cross-sectional survey of 3,222 children, young people and parents
Source: PLOS Glob Public Health. 2023 Apr 13;3(4):e0000963. doi: 10.1371/journal.pgph.0000963 (PMC10101632; doi:10.1371/journal.pgph.0000963)
Supplement: Attachment — Submitted filename: Response to Reviewers_Survey Publication.docx [file pgph.0000963.s017.docx]

**Response to reviewer comments:**

| **Editorial comments:** | |
| --- | --- |
| **Comment** | **Response** |
| Our staff editors have determined that your manuscript is likely within the scope of our Climate Change and Human Health Call for Papers. This editorial initiative is headed by a team of Guest Editors for PLOS GPH: Renzo Guito (St. Luke's Medical Center College of Medicine, Philippines) and Tolu Oni (University of Cambridge) as well as the Guest Editor for PLOS Climate Anna Stewart Ibarra (Inter-American Institute for Global Change Research). The Collection will feature research that addresses all aspects of the intersection between climate and health, from the changing burden of communicable and non-communicable disease to the impacts of extreme events on health systems, as well as research that assesses potential adaptations to build healthier and more resilient societies. Additional information can be found on our announcement page: [https://collections.plos.org/call-for-papers/climate-change-and-human-health/](https://protect-eu.mimecast.com/s/QsSzCl714TkQK5XuGDZUy?domain=collections.plos.org).  If you would like your manuscript to be considered for this collection, please let us know in your cover letter and we will ensure that your paper is treated as if you were responding to this call. Please note that being considered for the Collection does not require additional peer review beyond the journal’s standard process and will not delay the publication of your manuscript if it is accepted by PLOS GPH. If you would prefer to remove your manuscript from collection consideration, please specify this in the cover letter. | Thank you for bringing the Climate Change and Human Health Call for Papers to our attention. We would appreciate it if you would consider our manuscript for this collection, thanks! |
| Please provide additional details regarding participant consent. In the Methods section, please ensure that you have specified (1) whether consent was informed and (2) what type you obtained (for instance, written or verbal). If your study included minors, state whether you obtained consent from parents or guardians. If the need for consent was waived by the ethics committee, please include this information. | We have provided additional detail regarding the informed participant consent process in each instance consent was mentioned in the manuscript:  **Specifically, the following text was added at the following lines:**  Line 121 : “*...provided informed consent via an online form (c - S4 Appendix)…"*  Line 206: “*The number of social media users that clicked on the advertisement on either social media site and were successfully transferred to the survey landing page on Typeform.com to begin eligibility and informed-consent questions*  Additionally, a section on informed consent was added to the methods section of the manuscript. This section directs readers to the informed consent questions found in the supplementary materials:  **Specifically, the following text was added at line 114:**  *“Informed consent was gained from all survey respondents. This was through completion of an online form as approved by the London School of Hygiene and Tropical Medicine (LSHTM) Ethics Committee (see S4 Appendix for consent questions). Given the data collected by the survey was exclusively non-identifiable and would be instantly anonymous, the Ethics Committee agreed this research low risk. In agreement with the Ethics Committee, we did not seek to inform or obtain consent from parents or guardians of child respondents (who were all 13 years old or older).”* |
| Please send a completed 'Competing Interests' statement, including any COIs declared by your co-authors. If you have no competing interests to declare, please state "The authors have declared that no competing interests exist". Otherwise please declare all competing interests beginning with the statement "I have read the journal's policy and the authors of this manuscript have the following competing interests:" | **We have in included the following statement in the manuscript at lines 398:**  *“The authors declare no associations (financial or other-wise) with any commercial entities that have interests in the research completed in this manuscript. RCH has worked as a paid adviser to charities The Children’s Investment Fund Foundation and the Clean Air Fund.”*  For the reviewers, a separate ‘Competing Interests’ statement has been completed and submitted alongside the updated manuscript.  **Specifically, this statement includes the following text:**  *The authors declare no associations (financial or other-wise) with any commercial entities that have interests in the research completed in this manuscript. RCH has worked as a paid adviser to charities The Children’s Investment Fund Foundation and the Clean Air Fund.”* |
| Please amend your detailed Financial Disclosure statement. This is published with the article. It must therefore be completed in full sentences and contain the exact wording you wish to be published.  a. State the initials, alongside each funding source, of each author to receive each grant. b. If any authors received a salary from any of your funders, please state which authors and which funders.  If you did not receive any funding for this study, please simply state: “The authors received no specific funding for this work.” | We have amended the financial disclosure statement in our manuscript to include the initials of the author who received the grant funding and the authors whose salaries were funded (in-part) by the grant.  **Specifically, the following text was added at line 403:**  *“This work was supported by the Fondation Botnar [OOG-21-006] (received by RH and AD). This grant supported the salary (in-part) of all authors of this manuscript (RJ, SS, RP, JM, AB, SY, PW, AD, RH).”* |
| We have noticed that you have uploaded Supporting Information files, but you have not included a list of legends. Please add a full list of legends for your Supporting Information files after the references list. | Thank you for bringing this to our attention. We have added in a supplementary index to our “Supplementary Materials” document and have copied the index into the manuscript, after the references list. |
| Please send a completed 'Competing Interests' statement, including any COIs declared by your co-authors. If you have no competing interests to declare, please state "The authors have declared that no competing interests exist". Otherwise please declare all competing interests beginning with the statement "I have read the journal's policy and the authors of this manuscript have the following competing interests:" | Apologies for not including the attachment with the previous round of edits! The authors have declared that no competing interests exist – to confirm this, a separate ‘Competing Interests’ document has been completed and submitted alongside the updated manuscript. |
| 2. Please amend your detailed Financial Disclosure statement. This is published with the article. It must therefore be completed in full sentences and contain the exact wording you wish to be published.  a. Please clarify all sources of financial support for your study. List the grants, grant numbers, and organizations that funded your study, including funding received from your institution. Please note that suppliers of material support, including research materials, should be recognized in the Acknowledgements section rather than in the Financial Disclosure. b. State the initials, alongside each funding source, of each author to receive each grant. For example: "This work was supported by the National Institutes of Health (####### to AM; ###### to CJ) and the National Science Foundation (###### to AM)." | We have amended the financial disclosure statement in our manuscript to be in complete sentences:  **Specifically, the following text was added at line 398 now reads:**  *“The authors declare no associations (financial or other-wise) with any commercial entities that have interests in the research completed in this manuscript. RCH has worked as a paid adviser to the following charities: the Children’s Investment Fund Foundation and the Clean Air Fund.”*  **We have reviewed the funding statement at line XYZ and find it to be in line with the example you provided. Specifically, it reads:**  *“This work was funded by the Fondation Botnar [OOG-21-006 to RH and AD]. This grant supported the salary (in-part) of all authors of this manuscript (RJ, SS, RP, JM, AB, SY, PW, AD, RH).”*    **Additionally, we have provided acknowledgement for in-kind support at line 497:**  *“We would like to acknowledge and appreciate the support Meta has provided for this survey, in the form of advertisement credits used for recruiting participants to this survey.”* |
| 3. We note your current Data Availability statement as follows:  "All data collection tools, anonymised raw data, and data analysis code are available on the LSHTM Data Compass Site mirrored on Figshare. The dataset has been assigned a unique DataCite DOI."  Thank you for uploading your study's underlying minimal data set ([https://journals.plos.org/globalpublichealth/s/data-availability#loc-minimal-data-set-definition](https://protect-eu.mimecast.com/s/PxGACk714TorMl0H95p_r?domain=journals.plos.org)) to figshare.  At this time, please provide us with the relevant URLs, DOIs, or accession numbers that may be used for others to publicly access these data. | Please see the following URL for LSHTM internal data sharing website Data Compass: <https://datacompass.lshtm.ac.uk/id/eprint/2654/> and Figshare DOI: 10.6084/m9.figshare.21947432 |
| **Reviewer 1** | |
| 1. The writing has to be revised for the readability of general readers. For example, abbreviations such as CYPP, and AP first appear without any explanations, though the original meanings are found subsequently in the text. | Thank you for pointing this out, we’ve gone over the whole manuscript to ensure all abbreviations are written out in full in their first instance of use. |
| 2. The figures are in poor resolution. It is hard to read especially Figure 1.  Also, it would also be better if a table could be used to summarize the respondents' information in the 59 cities. | We apologize for the low-resolution figures. We have done our best to increase the resolution and hope it is sufficiently readable, if required, we’re happy to share a higher resolution version via email or similar.  We have added an additional table to the supplementary appendix 9 that features the demographics of respondents (age and whether the respondents are young people or parents/expectant parents) stratified by country. |
| 3. The main text reports the total number of young people, parents, and expectant parents respondents included in the survey data. However, it is better to report the distribution of numbers of different categories of respondents across the 59 cities. It may be that some of the cities have only a few parents included. | We have considered your recommendation alongside the recommendation made above (“Also, it would also be better if a table could be used to summarize the respondents' information in the 59 cities.”) and have included an additional table in supplementary appendix 9 which describes the distribution of demographic characteristics across the 59 cities. |
| 4. I wonder if some of the tables in the Supplementary Material can be moved to the main text. I believe they are more important to convey the findings rather than supporting material in the appendix. | Thank you for this suggestion. We have reviewed the location of figures in both the manuscript and supplementary material, but on reflection have and have decided not to make the manuscript longer by adding to it; we are conscious that longer manuscripts tend to be read less frequently, and we hope to maintain higher readership with a shorter publication, albeit with providing considerable additional detail in the supplementary information. |
| 5. The paper should put more effort in demonstrating the pioneering role of the survey. In lines 67-68 of the manuscript, it mentions that "the ideas and expectations of CYPP for improving urban air quality are under-explored". Then the authors cite a couple of papers without further detailed comparison with previous works. | Thank you; we agree and appreciate this suggestion - we've added in the following lines which compare our efforts to work published by “My City Too”, the “Urban Work Index”, “CYCLES”, and Sloam (2019).  **Specifically, the following text was added at line 61:**  *“Within the last five years, several reports have been published featuring youth voices, which sought to highlight the opinions young people have on their urban environments (8–11).In the My City Too (8), young Londoners shared their aspirations for the city based on their lived experience. Though this visionary piece provided compelling ideas, no systematic attempt was made to understand young people’s perception or experience of air pollution.*  *The Young Londoners’ Priorities for a Sustainable City (9) mapped key issues reported by the young people against the sustainable development goals (SDGs). Within goal 11 of the SDGs (Sustainable Cities and Communities), environmental protection emerged as a key theme. Though air quality ranked second as the most important environmental issue, no recommendations for addressing air pollution were sought from the young people.*  *The Urban Work Index (10) utilized more systematic methods to survey and rank cities according to how young people rated different areas of urban living. Though this index highlights the beneficial aspects of each city in its current state, the young people were not asked to consider the impact of air pollution on their ratings.*  *The CYCLES report (11) provides profiles of seven majors cities throughout the world that describe the sustainability challenges faced by their young residents. These challenges were reported in several highly polluting sectors, including food, transport, home and energy use. Three of the seven cities included pollution in their description of one of these three polluting sectors. This narrative strengthens the evidence on the impact air pollution has on urban living, though again, no recommendations were made by the young people.”* |
| **Reviewer 2** | |
| **Figures** | |
| It is difficult to see the attached Figure files due to its low resolution. Please change the attached Figure files in high resolution. | We apologize for the low-resolution figures. We have done our best to increase the resolution and hope it is sufficiently readable, but can also share higher-resolution figures directly if helpful. |
| **Introduction** | |
| 1. Line 58–60: The authors need to clearly describe which country and city it is. | The survey was open to any city globally, though advertisements were targeted to specific cities. The text at line 86 clarifies this:  **Specifically, the text reads:**  *“The survey was available to CYPP from any city in the world, though targeted recruitment (through paid social media advertisements – S1 Appendix) was directed to CYPP in 16 major cities (Bhubaneswar, Dar es Salaam, Dhaka, Free Town, Glasgow, Harare, Jaipur, Lahore, London, Los Angeles, Mexico City, Milan, Nairobi, Quezon City, Quito, Tamale). These cities were chosen to reflect a variety of global settings, population sizes, and levels of development in both the Global North and South, and to include the hosts of the 2021 Conference of Parties (COP) 26 Summit (Glasgow and Milan).”* |
| 2. I suggest that additional specific effect sizes of health effects such as disease burden and excessive mortality (or morbidity) caused by air pollution. | We have added in additional information about the risks of morbidity due to air-pollution exposure in across the life-course beginning at line 44  **Specifically, we’ve added the following text:**  *“In 2016, the reduction in life expectancy translated to 6.5 million premature deaths due to household and ambient air pollution (3). Those who are not killed by air pollution face increased risk of morbidity even before their birth, with maternal PM2.5 exposure linked to ~18% of global preterm births in 2017 (4). Due to air pollution, these additional risks continue throughout early child development, adolescence, and young adulthood. Air pollution exposure has been associated with lung development and function in children, poor respiratory health in adolescence, and increased risk of lung cancer throughout the life course (4).”* |
| 3. I propose adding a rationale for the importance of urban air pollution compared with non-urban areas (e.g., rural areas). | We have clarified the importance of air pollution in urban areas at line 51  **Specifically, we’ve added the following text:**  *“Though young people in both urban and non-urban places are impacted by air pollution, young people in urban areas are exposed to significantly higher levels than those in rural areas. In addition, in many settings (particularly low- and middle-income countries), as urbanisation accelerates, air pollution increases (4).”*  However, we’ve provided further rationale for the urban-focus of this research in the subsequent lines 54  **Specifically, we have maintained the following text:**  *“Children currently represent 30% of the four billion people living in cities, but this will rise to 70% of 6.7 billion by 2050 (5). Additionally, many of the sources of AP contribute to climate change, the effects of which children today will experience throughout their lives (6).”* |
| **Methods** | |
| 1. Line 86–88: What is the basis for the age definition corresponding to the inclusion criteria? I suggest that the authors will add references or sentences. | Our inclusion criteria were guided by international regulations on access to social media, and widely used definitions of ‘youth’. However, in the absence of a global consensus/regulation on this, we, in conjunction with our youth advisers, took a pragmatic decision to globally include young people aged between 13 and 25. We’ve clarified this at line 94  **Specifically, we have added the following text:**  *“A child was differentiated from a young person at the age of 13 due to internet regulations in most countries which require children under the age of 13 to seek parental approval for most internet use. Young people included those up to the age of 25. Though this is the standard age in many countries, it is not a globally recognised number. As this was a global survey, this cut-off point was selected by the research team in conjunction with youth advisers for the project.”* |
| Data collection | |
| 1. Line 91–92: I wonder if the study participants were voluntary participation or there was no benefit. | Participation was voluntary, and nor remuneration of gifts in kind were provider. This, and the rationale for it, is explained in the supplementary methods material. In the consent questions of supplementary 4, statements are included about the voluntary nature of the survey and the absence of reward. Young people needed to confirm they understood these statements in order to complete the survey. We have clarified this point in the body of the manuscript and directed readers to this supplementary material.  **Specifically, we have added the following text at line 103:**  *“Participation in this survey was voluntary and respondents were informed they would not receive a reward for completion (for full statements, see S4 Appendix).”* |
| 2. I suggest that an indicator has been added to present the reliability and validity of the constructed questionnaire (e.g., Cronbach’s alpha). | Thank you for this suggestion. We have consulted our statistician and have determined our data doesn’t meet the conditions required for the use of Cronbach’s alpha. For example, we do not assume our data to be normally distributed.  To assess validity and reliability, we completed several rounds of pretesting of the survey instrument with a group of youth advisors and amongst members of our team.  We have included the following text about pre-testing methods (used for reliability and validity assessment) in Supplementary Appendix 2:  *“The English-language survey instrument was pre-tested in two phases to evaluate its reliability and validity and ensure data quality.  Within each phase of pre-testing, feedback on the survey instrument was collected using a Google Form (25), in parallel time with the completion of the survey instrument. Feedback was integrated into the draft Typeform survey instrument after each phase of pre-testing.”*  We’ve also added the following text into Supplementary appendix 2 to communicate the readability and interpretability of the questionnaire (for the English language version of the survey; we have not done this for all translations).  *“The reliability of the English-language survey instrument was further tested using two different scoring systems for readability and interpretation. The survey scored 82.3 out of 100 for the Flesch Reading Ease score which indicates good overall readability. To assess the ease of interpretation for young people who have varying language comprehension levels, the CEFR Level was tested and rated as B2, sufficient for those with conversational English abilities.”* |
| Missing data | |
| 1. Line 119–120: The authors need to specify which imputation method was used. For the robustness of the research results, I suggest to compare the results before and after using the imputation method. | We have clarified the imputation methods in the subsequent sentences.  **Specifically, the text now reads:**  *“Where possible, imputation methods were used to limit non-response bias for age and location using the following methods. Where age was not reported or reported as an unrealistic value in excess of 100 or less than 13 years old, age was recoded as ‘missing’ and survey respondents were analysed together. Where location was not reported or only reported by region or country, probable location was imputed based on the city featured in the recruitment advert clicked on.”* |
| Data analysis | |
| The authors need to clearly explain the data analysis. | Thank you for your comment. The data analysis plan was published on Data Compass (an LSHTM-based server), explained in brevity within the manuscript starting at line 134, and described in detail in the supplementary appendix 2.  **We have added the following to the main manuscript:**  *“In short, descriptive statistics were completed for the full sample and subpopulations by city and for each survey question. The quantitative and qualitative survey responses were analysed for the full sample and results were stratified by age, whether the respondent was a young person or parent/expectant parent, and by the PM2.5 pollution in their city.”* |
| Results | |
| 1. Line 185–187: On a typical person basis, how many minutes does the questionnaire take? If the response is too fast, or too slow, you may get distorted results. I propose to present the distribution of survey response time. In addition, I am curious about the analysis results after excluding both extreme values of the survey response time. | We have included the following text at line 183 to clarify on this point:  **Specifically, the text now reads:**  *“The survey was built to take less than 10 minutes to complete, however, Typeform allows respondents to leave and return to a survey an unlimited number of times using the same link prior to submission. Subsequently, the median survey completion time was 8.7 minutes (range: 0.5 - 458.8 minutes). Responses were not excluded from the analysis for outlying times”* |
| 2. Line 192–192: I suggest that the average age is presented by separating children and young people | Beginning line 192, we have added the mean age and SD for each target group (young people, parents, and expectant parents). The age of children less than 13 years old is not directly measured, as the parents respond on their behalf and report their own age (see supplementary 3 for survey questions). We hope the distribution of age by target population will suffice.  **Specifically, the text now reads:**  *“75% of all respondents (n=2,430) were young people aged 13-25 years old (mean age 21, SD 4.3); 16% (n=509) were parents of children aged <13 (mean age 39, SD 7.8); and 9% (n=283) were expectant parents (mean age 32, SD 7.3)”* |
| Discussion | |
| 1. Line 363-385: I suggest that the authors compare previous studies by survey year, country or continent, race. | Thank you for your suggestion, which we have discussed amongst authors. We feel that, given the relative paucity of literature on this area, it is clearer to organize references to previous studies following the order of the survey itself, allowing us to highlight similarities in a sequence that follows our results section. |
| 2. Air pollution levels and economic levels will be different in Europe, the United States, Asia, Africa and etc. The level of awareness of air pollution will vary depending on the social infrastructure and urbanization, including race, socioeconomic level, developing and developed countries. In the future, I hope that research will be conducted in more countries and global study will be conducted, and I suggest that related sentences be mentioned in Discussion. | Thank you this this suggestion, we completely agree - these are future areas we would hope to see included on research agendas.  **We’ve added the following text at line 348:**  *“Future research into this issue could employ better methods to improve sample representativeness (e.g. combining online and offline surveying with particular efforts to include marginalised communities (e.g. homeless and migrant youth and those in informal settlements)) as air pollution levels and youth’s awareness of air pollution will differ depending on the social infrastructure, and other determinants. Further research on the experience of urban youth is needed across all socio-economic levels, races, and must include cities of all economic statuses and levels of urbanization.”* |
